# Improving AlphaFold predicted contacts in alpha-helical transmembrane proteins structures using structural features

**DOI:** 10.21203/rs.3.rs-3475769/v1

**Published:** 2023-10-26

**Authors:** Aman Sawhney, Jiefu Li, Li Liao

**Affiliations:** 1 Department of Computer and Information Sciences, University of Delaware, Smith Hall, 18 Amstel Avenue, Newark, DE, 19716,United States.; 2 School of Optical-Electrical and Computer Engineering, University of Shanghai for Science and Technology, 516 Jun Gong Road, Shanghai 200093, P. R. China.

**Keywords:** Protein structure, Alpha helix, Contact map prediction, Transmembrane proteins, Protein structure modeling, Machine learning, Alphafold

## Abstract

**Background:**

Residue contacts maps offer a 2-d reduced representation of 3-d protein structures and constitute a structural constraint and scaffold in structural modeling. In addition, contact maps are also an effective tool in identifying interhelical binding sites and drawing insights about protein function. While most works predict contact maps using features derived from sequences, we believe information from known structures can be leveraged for a prediction improvement in unknown structures where decent approximate structures such as ones predicted by AlphaFold2 are available.

**Results:**

Alphafold2’s predicted structures are found to be quite accurate at inter-helical residue contact prediction task, achieving 83% average precision. We adopt an unconventional approach, using features extracted from atomic structures in the neighborhood of a residue pair and use them to predicting residue contact. We trained on features derived from experimentally determined structures and predicted on features derived from AlphaFold2’s predicted structures. Our results demonstrate a remarkable improvement over AlphaFold2 achieving over 91.9% average precision for held-out and over 89.5% average precision in cross validation experiments.

**Conclusion:**

Training on features generated from experimentally determined structures, we were able to leverage knowledge from known structures to significantly improve the contacts predicted using AlphaFold2 structures. We demonstrated that using coordinates directly (instead of the proposed features) does not lead to an improvement in contact prediction performance.

## 1 Introduction

Transmembrane (TM) proteins are involved in a wide range of critical cell processes such as signal transduction, transport of ions & molecules across membranes, catalysis and protein targeting [1]. In the event of dysregulation of cellular function, manipulation of these processes via therapeutic interventions can restore homeostatis [2]. It is therefore no surprise that 60% of all clinically approved drugs target membrane proteins [2].

About 20 to 30 percent of genes in all genomes encode membrane proteins [3, 4]. Further, TM proteins are predominantly α-helical [5]. There exists a dramatic gap between protein sequences and experimentally determined structures, especially in the case of TM proteins [6]. Since the extraction of membrane proteins from their native lipid environment can alter their integrity and their hydrophobic nature resists water dissolution, preventing crystallization which is essential for techniques like X-ray crystallography [7, 8]. Though there have been several advances, such as attempts to map the structure while embedding in a lookalike lipid membrane [9] and making them water-soluble [10], the number of solved structures remains disproportionately low. The 3-d structure of TM proteins is fundamental in understanding their function and to aid drug design [8]. In the absence of 3-d structures, residue contact map offers a reduced 2-d representation of 3-d protein structure which is translation and rotation invariant and, is easily ingested by learning models. Generating a 3-d protein model using 2-d contact maps is an actively researched problem. Typically, a folding engine like Rosetta [11] is used which uses binary contact maps as geometric constraints and turns them into folded proteins [12]. But residue contact predictions themselves have been used for protein-protein interaction prediction [13, 14], accelerating molecular dynamics simulations [15] and in predicting binding affinity in docking simulations [16]. TM helices have been observed to tilt and bend when protein structure captured in different functional states are compared [17]. Hence, a 2-d contact map can be an effective tool in itself, to identify inter-helical binding sites, thus providing insights about a protein’s functions.

Various features based on sequence, physio-chemical properties and co-evolutionary information [6] have been used in literature to predict residue contacts. Evolutionary coupling (EC) approaches, such as direct coupling analysis [18] and EVFold [19], compute residue pair co-evolutionary propensities (which correlate with contact propensities) from multiple sequence alignments (MSAs) and have proved more effective than others. Several methods employed supervised learning to combine predictions from various EC methods as input features to improve performance. These included deep learning approaches such as DeepMetaPSICOV [20], Wang et al. [21] and Deep-Helicon [6]. It has also been reported that topological patterns in the neighborhood of a residue pair in the contact map, such as contact propensity of the surrounding positions, can further enhance the prediction accuracy [22].

The use of residual networks (ResNets) [23] with Convolutional neural networks (CNNs) greatly improved the quality of the predicted contact maps [12]. Raptor X [21], AlphaFold [24] and TrRosetta [25] all used ResNets for residue contact prediction with great success. An updated RaptorX system [26], predicted discretized inter-residue distances (1.5*A*° increments) instead of binary contacts. AlphaFold [24] employed a similar technique, and added components to convert predicted distribution over distances into smooth energy potentials that could be minimized using gradient descent and folded into a 3-d structure without the use of a folding engine [12]. These approaches input MSAs directly and employed a deep learning pipeline to predict residue 3 -d coordinates, providing an increasingly effective end to end solution. Recently, Alphafold2 [27] with the use of transformers [28] was able to achieve near-angstrom accuracy given sufficiently deep MSAs [12].

Given the great success of AlphaFold2, it is conceivable to question whether any other efforts in structural prediction including contact map prediction have become superfluous. Several studies have examined AlphaFold2’s predicted structures, for example to assess the impact of conformational diversity on its predictions [29] or to evaluate if AlphaFold2 learned the physics of folding [30]. In particular, TmAlphaFold [31] examined if AlphaFold2’s predicted alpha-helical TM structures are realistic. They found the quality for a majority of cases (out of 215,844 TM proteins) to be excellent (45.16%) or good (21.51%) and for a lower proportion of proteins, the quality to be fair (25.08%) or poor (2.21%). AlphaFold2 self reports an all-atom accuracy of 1.5*A*° r.m.s.d.95 (95% confidence interval = 1.2–1.6 *A*°) [27].

Despite AlphaFold2’s high accuracy, there is room for improvement, especially for TM proteins. MULTICOM3 [32] is built on top of AlphaFold2 and AlphaFold-Multimer [33]. It improved upon AlphaFold2’s performance by sampling more structural models via adjustment of input MSAs and incorporating protein complexes. CGAN-Cmap [34] used a generative adversarial neural network embedded with a series of modified squeeze and excitation residual networks to predict residue contact maps on CASP datasets and achieved a performance gain over contact maps extracted from AlphaFold2.

As it comes to contact map prediction, our previous work shows that information from existing 3-d structures could be leveraged to improve prediction accuracy [35]. A classifier trained on structural features extracted from a residue pair’s neighborhood was found to significantly outperform state-of-the-art models using non-structural features, achieving above 90% precision for top L/2 and L inter-helical contacts. In particular, those structural features were also found to be robust to high levels of noise, pessimistically reliable up to 2*A*° of coordinate noise [35]. It is then intriguing to explore the possibility of applying this idea of using structural features for contact prediction to proteins that do not have experimentally determined structure but only have decently approximate structures predicted by a computational tool such as AlphaFold2. Here we explore this idea expanding on our previous work. While AlphaFold2 is not designed for contact map prediction per se but rather for tertiary structure as a whole, its predicted structure nonetheless can be used to establish a contact map as a by-product. And therefore we hypothesize that a general purpose tertiary structure prediction tool like AlphaFold2 can be “bootstrapped” with features extracted from its predicted structure to perform better for some special purpose tasks such as contact map prediction.

In this work, we set to advance the use of structural features to enhance AlphaFold2’s performance for contact map prediction, although AlphaFold2’s performance is typically measured for 3-d structures, in terms of predicted local distance difference test (pLDDT) [36]. As previously explained, contact maps are useful on their own, hence we first evaluate how well AlphaFold2’s predicted structure can deliver for contact point prediction. We found it to be already quite accurate, achieving over 83% average precision for the held-out datasets. We then trained a neural network based classifier on structural features derived from experimentally determined structures and applied the trained classifier to predict residue contacts for proteins with derived features from AlphaFold2’s predicted structures. The results from our experiments show that this method achieved over 91.9% average precision for for the held-out datasets, significantly outperforming AlphaFold2 predictions. Furthermore, we compared our derived structural features with using 3-d coordinates directly, and found that the latter approach fails to improve upon AlphaFold2 predictions.

## 2 Materials

### 2.1 Dataset - Experimentally determined structures

We adopt the widely used DeepHelicon dataset [6] for this study. The dataset was constructed using 5606 α-helical TM proteins chains from the PDBTM database [37], each with resolution better than 3.5*A*°. It was made non-redundant at 23% sequence identity level and with a maximum TM score [38] of 0.4 to ascertain that the protein chains were structurally dissimilar. The dataset consists of a total of 222 protein chains, with a varying number of TM helices (2–17). It is divided into three sub-datasets a) TRAIN - a training set of 165 sequences b) TEST - a held out set of 57 sequences and c) PREVIOUS - a held out set of 44 sequences [39, 40]. For every protein chain, the dataset contains the protein sequence, annotations of which residue pair positions are contacting, which residue positions are in the TM zone, and the 3-d coordinates for heavy atoms of each residue in PDB atomic coordinate format [41]. DeepHelicon’s model predictions for the TEST and PREVIOUS datasets are included as well.

Given a chain’s atomic structure, a residue pair is considered to be in contact if the distance between their heavy atoms is below a certain threshold. DeepHelicon dataset [6] defines two residues to be in contact (contact point), if the least distance between any pair of their heavy atoms is less than 5.5*A*° and if they are sequence separated by a minimum of 5 residues [6].

Following our previous work [35], a few sequences are removed - those are sequences with no inter-helical contact points or with positions annotated to be in TM zone not matching positions used by DeepHelicon (refer to the Supplementary file 1) - leaving a total of 162 sequences in the TRAIN dataset, 40 sequences in the PREVIOUS dataset and 54 sequences in the TEST dataset. These changes for all datasets are summarized in Table 1.

### 2.2 Dataset - Alphafold predicted structures

AlphaFold DB provides predicted structures for over 200 million protein sequences in the UniProt [42] reference proteome [36, 43]. These structures can be accessed via the protein chain’s UniProtKB ID [42], and the 3-d coordinates for each residue’s heavy atoms are available in PDB atomic coordinate format. We relied on Research Collaboratory for Structural Bioinformatics protein data bank (RCSB PDB ^1^) [41] to map the PDB ID of every chain in the DeepHelicon dataset to UniProtKB ID. If a match was found, the corresponding predicted structure was accessed via AlphaFold DB. For several protein chains, an integer offset to PDB positions in the DeepHelicon dataset is needed to sequentially align them with Alphafold structures (refer to Supplementary file 1). In case a UniProtKB ID match was not found in RCSB PDB or the sequences from UniProt and DeepHelicon dataset matched partially i.e. all positions annotated to be in TM zones were not contiguously included, then the chain was removed from the dataset (refer to the Supplementary file 1). This process leads to a final total of 154 sequences in the TRAIN dataset, 34 sequences in the PREVIOUS dataset and 49 sequences in the TEST dataset.

These changes and contact ratio (CR) (for residue pair positions of interest - within different TM zones and sequence separated by a minimum of 5 residues), for all datasets are summarized in Table 1, where CR is defined as equation 1.


(1) $$CR=\frac{\#\mathrm{contact}\;\mathrm{points}}{\#\mathrm{residue}\;\mathrm{pair}\;\mathrm{positions}}$$

As mentioned in section 2.1, DeepHelicon dataset includes annotations indicating residue positions located in the TM zone. For matching structures obtained from AlphaFold DB, we adopt the same annotations. Following the contact definition described in Section 2.1, for matching predicted structures obtained from AlphaFold DB, we generated annotations indicating which residue pair positions are contact points.

## 3 Methods

The methods proposed for predicting residue contact maps consist of mainly two parts: selecting features and training a classifier. In the following, we show in details how to construct a feature vector from a 3-d structure, either experimentally determined or computationally predicted, to represent a residue pair, and how to use them to feed into a neural network based classifier for training.

### 3.1 Structurally derived features (SDF)

**Fig. 1:**
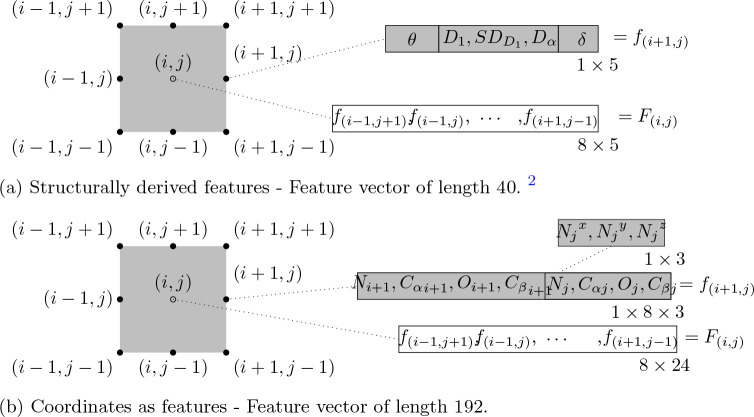
Neighborhood feature vector - Features extracted from a 3 × 3 neighborhood window (excluding the center) around a residue pair of interest (i,j).

Following our previous work [35], we employ structural features derived from coordinate data for residue contact prediction. Only residue pairs (i,j), where i, j are amino acid sequence positions, s.t. |i−j|>5 and i and j are on separate helices (interhelical) are considered. For each of the eight positions in the neighborhood window of size 3 × 3 around (i,j) (excluding the center (i,j)), a feature vector consisting of the inter-helical tilt angle, relative residue distances and relative residue angle (length 5) is constructed. We concatenate features for these eight neighboring positions to obtain a feature vector of length 40 (8 × 5). This process is illustrated in Figure 1a. In the following sub sections, we describe the extracted features in some detail.

#### Inter-helical tilt angle (θ)

For a residue pair, inter-helical tilt angle is defined as the angle between the helices the residues reside on [44]. In an α-helix, each main-chain C=O and N−H group is hydrogen bonded to a peptide bond four residues away i.e. O(i) to N(i+4) (where i is the $i^{th}$ residue). The peptide planes are roughly parallel with the helical axis and the dipoles within the helix are aligned, i.e. all C=O point in the same direction and all N−H point in the other direction, while the side chains point outward from the helical axis (generally oriented towards the amino-terminal) [45]. Motivated by this observation, we compute any helical axis orientation by averaging the direction of C(i)=O(i)−N(i+4) for all residues in the helix. The angle between the axes of two helices is the inter-helical tilt angle. We use the Pymol package for these computations [46–48]. A diagrammatic representation is provided in Supplementary file 1.

#### Relative residue distance

We define three relative residue distance features:

1. $D_{1}$ distance (mean relative residue distance) [35, 49, 50] - For any pair of residues, we compute the mean Euclidean distance between all pairs of their heavy atoms. Let $\left\{A_{x}^{1},\ldots A_{x}^{M}\right\}$ and $\left\{A_{y}^{1},\ldots A_{y}^{N}\right\}$ be the euclidean (3-d) coordinates of the atoms of residues $R_{x}$ and $R_{y}$ respectively. Further, let dist(i,j) denote the Euclidean distance between two 3-d coordinates i and j then mean residue distance between $R_{x}$ and $R_{y}$ is
(2) $$D_{1}\left(R_{x},R_{y}\right)=\frac{1}{MN}\sum_{i=1}^{M}\sum_{j=1}^{N}dist\left(A_{x}^{i},A_{y}^{j}\right)$$
2. $D_{1}$ deviation (relative residue distance deviation) [35, 49, 50] - For any pair residues, we compute the standard deviation of the euclidean distances between all pairs of their heavy atoms. So relative residue distance deviation between residues $R_{x}$ and $R_{y}$ is
(3) $$SD_{D_{1}}\left(R_{x},R_{y}\right)=\sqrt{\left\{\frac{1}{MN}\sum_{i=1}^{M}\sum_{j=1}^{N}[{\left(dist\left(A_{x}^{i},A_{y}^{j}\right)-D_{1}\left(R_{x},R_{y}\right)\right)}^{2}]\right\}}$$
3. $D_{\alpha }\;(C_{\alpha }$ distance) [35, 49, 50] - For any pair of residues, we compute the euclidean distance between their alpha carbons. Let atom(R,k) be a function that returns the $k^{\text{th}}$ atom for residue R. Further, let $atom\left(R_{x},i\right)=C_{\alpha }$ and $atom\left(R_{y},j\right)=C_{\alpha }$ then
(4) $$D_{\alpha }\left(R_{x},R_{y}\right)=dist\left(A_{x}^{i},A_{y}^{j}\right)$$

#### Relative residue angle (δ)

We defined a residue’s plane as formed by the vector between $C_{\alpha }$ and N atom and the vector between $C_{\alpha }$ and C atom of the carboxyl group [50]. For a residue pair, we define the relative residue angle as the absolute angle between the surface-normals of the residue planes [35]. A diagrammatic representation is provided in Supplementary file 1.

Note that the definition of residue contact is based on minimum distance between the residue pair’s heavy atoms, while we use residue pair’s neighborhood structural information (distance functions used are different as well) to predict whether it is in contact.

### 3.2 Coordinates as features (CF)

To demonstrate the effectiveness of our derived features described above, we also directly use 3-d coordinates of residue pair’s heavy atoms as features. This serves as a performance baseline. For a residue pair position (i,j), where i, j are amino acid sequence positions, s.t. |i−j|>5 and i and j are on separate helices (inter-helical), we select a neighborhood window of size 3 × 3 around it. For each of the eight positions around (i,j) (excluding the center (i,j) ), we construct a feature vector of length 24 - consisting of x, y, z coordinates of the heavy atoms from the residue pair of interest. Each residue is represented by the x, y, z coordinates of 4 heavy atoms from its structure namely - Nitrogen atom (N) from the amino group, alpha Carbon $\left(C_{\alpha }\right)$, Oxygen atom from the carboxyl group (O) and beta Carbon $\left(C_{\beta }\right)$. We concatenate features for these eight neighboring positions to construct a feature vector of length 192 (24 × 8). This process is illustrated in Figure 1b.

### 3.3 Classification experiment

We handled the prediction of an inter-helical TM residue pair position being a contact point as a supervised binary classification problem. As stated previously, only residue positions that are sequence separated by at least 5 residue positions were considered. For structurally derived features, we constructed a feature vector of length 40 (described in Section 3.1). While using coordinates as features, a feature vector of length 192 was formed (described in 3.2).

Features from either feature set (structurally derived or coordinates) were first normalized to a [−1, 1] scale before being used for classification, such that $f_{i_{\mathrm{scaled}}}^{t}=-1+2\times \left(\frac{f_{i}^{t}-min\left(f_{i}\right)}{max\left(f_{i}\right)-min\left(f_{i}\right)}\right)$ where $f_{i}^{t}$ is the $t^{th}$ sample for the feature $f_{i},$ max(.) and min(.) compute the maximum and minimum observed value for the feature $f_{i}$ and $f_{i_{\mathrm{scaled}}}^{t}$ represents the scaled value of $t^{th}$ sample for the feature $f_{i}$.

**Fig. 2:**
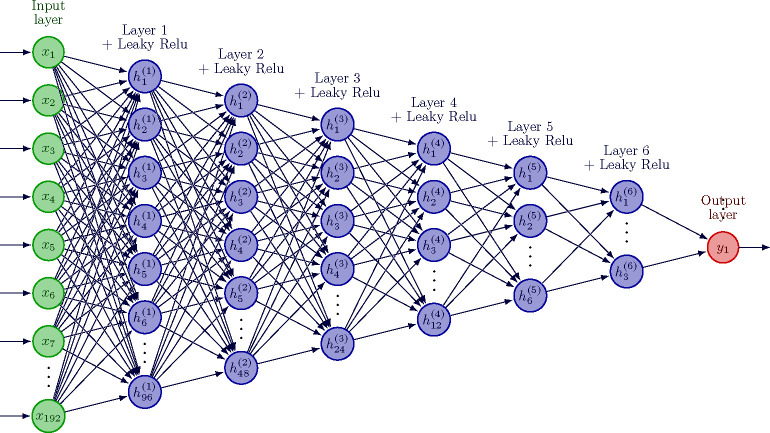
Neural network architecture ^3^

We constructed a neural network classifier consisting of 6 hidden layers with leaky Relu activation function [51] to capture the non-linearity in the features and used binary cross entropy as the loss criterion at the output. The architecture is depicted in Figure 2. Using Adam optimizer [52] with a learning rate of 0.0001, we trained in batches of 256 samples for a total of 400 epochs. The weights of the network were initialized using Xavier uniform distribution [53] and gradients were clipped to the range [−1, 1] to prevent exploding and vanishing gradients [54]. We used PyTorch package for our implementation [55].

A static fully connected linear layer was used to project structurally derived features from 40 to 192 dimensions, this enabled us to use the same network for both (structural derived and coordinates) feature sets.

We assessed our performance on each dataset - TRAIN (154 sequences), TEST (49 sequences) and PREVIOUS (34 sequences) using 5 fold cross validation[56]. In each fold, 80% of randomly selected training sequences were used for training and 20% were held out for validation. Further, we retrained on the full TRAIN dataset and evaluate the performance on held out the PREVIOUS and TEST datasets.

In each experiment, we used features (SDF or CF) constructed from experimentally determined structures during training and, for comparison purpose, tested the trained classifier on two separate cases: a) features constructed from experimental determined structures, and b) features constructed from Alphafold predicted structures.

#### Performance metrics

We evaluated the classification performance with the following two widely used metrics:

1. AUC-ROC - The area under the Receiver operating characteristic curve computed using the trapezoidal rule. [57, 58]
2. Average precision - Average precision summarizes the precision recall curve as weighted mean of precision at each threshold, wherein the increase in recall from the previous threshold is used as the weight. [58]
(5) $$Average\;Precision\;=\sum_{n}\left(R_{n}-R_{n-1}\right)P_{n}$$
  where $P_{n}$ and $R_{n}$ are precision and recall at the $n^{\mathrm{th}}$ threshold. For predicted structures from Alphafold DB, we generate binary annotations for whether a residue pair is a contact point (described in Section 2.2). In equation 5, this is the case when there is only one (n=1) threshold and, AveragePrecision=P×R; where P and R are the observed precision and recall scores using these binary labels.

These two metrics allow us to evaluate a classifier’s predictive power without imposing a threshold on the prediction score so that an overall assessment can be achieved, not tied to a specific threshold choice. Once the test examples are ranked by their prediction score from a classifier, an ROC curve can be plotted the true positive rate as a function of false positive rate by running down the ranked list as follows: a) at each position in the list, predict the test examples above as positive and below as negative, b) compare the prediction with the ground truth label to determine true positive and false positive, and c) calculate the rates and move to the next position in the list. The higher the curve – more true positives predicted at a given false positive rate, the better the performance, which is measured as the area under the curve, a value (called ROC score) between 0 and 1, with 1 being the perfect performance and 0.5 being a performance comparable to a random toss-up. Using a similar procedure running down the ranked list of test examples, a curve can be plotted with precision as a function of recall. Average precision is essentially the area under the precision-recall curve. It has been reported [59] that for skewed data – with a much larger proportion of negative examples, which is our case, ROC scores tend to be more optimistic than the actual performance is and, for such cases, average precision may presents a more realistic picture. For both metrics, we report the mean value across all sequences.

## 4 Results

### 4.1 Classification

We report the average 5 fold cross validation performance (repeated 5 times) from using both feature types - CF and SDF, and from AlphaFold2 binary annotations and DeepHelicon predictions, as measured in terms of Average Precision and AUC-ROC, in Table 2.

The performance of either feature type (Table 2) when constructed using experimentally derived structures is an upper bound. Upper bound performance using our SDF substantially exceeds using coordinates directly (CF) by 11.22%, 12.59% & 13.89% for TRAIN, TEST and PREVIOUS datasets in terms of Average precision; 0.72%, 0.87% & 0.71% in terms of AUC-ROC.

SDF constructed using AlphaFold2 predicted structures (SDF + AF) outperforms AlphaFold2 binary annotations by 10.36%, 7.95% & 5.65% for TRAIN, TEST and PREVIOUS datasets respectively in terms of average precision; 4.78%, 3.96% & 4.72% respectively in terms of AUC-ROC. Further, SDF + AF comfortably outperforms DeepHelicon by 34.32%, 33.6% for TEST and PREVIOUS datasets respectively in terms of average precision; 6.20% & 6.00% respectively in terms of AUC-ROC.

We report the average classification performance for the held out datasets (TEST & PREVIOUS) for both feature types - CF and SDF, AlphaFold2 binary annotations and DeepHelicon predictions in Table 3. The upper bound performance using our SDF substantially exceeds using Coordinates directly (CF) by 14.77% & 15.51% for TEST and PREVIOUS datasets respectively in terms of Average precision; 0.79% & 0.68% respectively in terms of AUC-ROC.

SDF+AF outperforms AlphaFold2 annotations by 9.5% & 7.25% for TEST and PREVIOUS datasets respectively in terms of average precision; 3.96% & 3.25% respectively in terms of AUC-ROC. SDF + AF comfortably outperforms DeepHelicon as well by 35.89%, 35.19% for TEST and PREVIOUS datasets respectively in terms of average precision; 6.22% & 6.02% respectively in terms of AUC-ROC. Further, in nearly all sequences, 98% of TEST and 97.1% of PREVIOUS datasets (Table 4), the classification performance is improved (measured in terms of Average Precision).

In all experiments, SDF outperforms the baseline CF. The classifier is trained on features constructed from experimentally derived structures. However during testing, only features constructed from AlphaFold predicted structures will be available to us. Consequently, classifier’s testing performance depends on whether the feature distributions from the two data sources (experimental vs AlphaFold prediction) are similar. In Table 5, we report the feature mean - average across all features and samples and feature variance - standard deviation across all features and samples, for TRAIN, TEST and PREVIOUS datasets. The datasets were first scaled to a range of [−1, 1]. It can be seen that SDF constructed using structured predicted by AlphaFold or experimentally determined structures are very similar, differing by 0.013, 0.017, 0.035 for TRAIN, TEST & PREVIOUS datasets respectively in terms of feature mean; −0.005, 0.003, −0.005 respectively in terms of feature variance. CF constructed using AlphaFold structures or experimentally determined structures vary more, differing by 0.207, 0.380 & 0.014 for TRAIN, TEST & PREVIOUS datasets respectively in terms of feature mean; 0.142, 0.067, & 0.049 respectively in terms of feature variance. These statistics are helpful in gauging the distribution similarity. Using relative residue distance and angles then potentially has the effect of scaling - mean removal and variance scaling. In other words, it is likely that an efficient model would need to predict relative angles and distances that are closer in distribution to experimental determined ones, hence SDF are a natural fit. CF’s exhibit higher variance from generated using experimentally determined structures, which makes intuitive sense as one would expect real residue coordinates to exhibit more variance than predicted ones.

We further examine this divergence of two data sources via a second auxiliary classifier’s ability to differentiate between features generated using the two sources (AlphaFold & Experimental) in Supplementary file 1. The results support what the simple statistics (means and variance) have revealed.

The contact prediction performance for held out datasets (TEST and PREVIOUS) is higher than corresponding cross validation experiments. We attribute this to a bigger training set size. Performance comparison for individual sequences, Precision and Recall scores for the top L, L/2, L/5, L/10 residue pair predictions where L denoted the total concatenated length of the TM helices for a sequence are reported in Supplementary file 1.

DeepHelicon dataset consists of structures that were experimentally determined prior to the release of AlphaFold DB, it is likely they were part of AlphaFold’s training, which then bolsters our case.

## 5 Conclusion

In this work, we developed an unorthodox approach of using features extracted from atomic structures in the neighborhood of a residue pair and used them for improving the prediction of inter-helical residue contact in α-helical TM proteins. This approach, which is in contrast to most other works that have focused on developing methods to predict residue contact based on primary structure, would not be useful should the atomic structures be not available. What we demonstrated here is that AlphaFold2 has dramatically raised the quality of predicted structures – in our held out experiments, we found it to be highly accurate achieving over 83% average precision – can be used as surrogate of ground truth 3d structure. We trained on features generated from experimentally determined structures and predicted on features constructed using AlphaFold2 predicted structures. The results from our experiments demonstrate a significant improvement over AlphaFold2, achieving over 91.9% average precision for both TEST and PREVIOUS datasets. Further, we demonstrate that simply training on coordinates directly does not lead to a performance improvement. Structurally derived features potentially reduce distributional distance between features derived from experimentally determined and predicted structures. This work demonstrates that residue sequence neighborhood is information rich, can be used to produce more accurate structures and that features derived from residue’s structural neighborhood are generalizable across sequences. As future work, it is conceivable that we may leverage the improved contact map to enhance predicted structures even further.

## Figures and Tables

**Table 1:** Dataset statistics - Number of protein chains and contact ratio for TRAIN, TEST and PREVIOUS datasets.

Dataset	#Sequences	#Filtered Sequences	AF available	*CR* × 100

TRAIN	165	162	154	2.10
TEST	57	54	49	2.07
PREVIOUS	44	40	34	1.95

AF available - A matching AlphaFold predicted structure was found

CR - Contact ratio

**Table 2:** Classification performance - average over 5 folds Cross validation (repeated 5 times).

Classifier	Structure source	Feature type	TRAIN		TEST		PREVIOUS	
			Average Precision	AUC-ROC	Average Precision	AUC-ROC	Average Precision	AUC-ROC

NN (ours)	Exp.	SDF	0.9569±0.0039	0.9980±0.0004	0.9497±0.0054	0.9981±0.0004	0.9456±0.0043	0.9983±0.0002
NN (ours)	AF	SDF	0.8956±0.0171	0.9919±0.0035	0.9111±0.0204	0.9957±0.0016	0.9038±0.0270	0.9965±0.0011
NN (ours)	Exp.	CF	0.8447±0.0193	0.9908±0.0018	0.8238 ±0.0341	0.9894±0.0041	0.8067±0.4712	0.9912±0.0035
NN (ours)	AF	CF	0.8125±0.0246	0.9846±0.0046	0.8349±0.0287	0.9915±0.0017	0.8254±0.0295	0.9927±0.0014
AlphaFold2	-	-	0.7920	0.9441	0.8316	0.9561	0.8473	0.9643
DeepHelicon	-	-	-	-	0.5679±0.0440	0.9337±0.0183	0.5678±0.0479	0.9365±0.0170

**Table 3:** Classification performance - held out datasets.

Classifier	Structure source	Feature type	TEST		PREVIOUS	
			Average Precision	AUC-ROC	Average Precision	AUC-ROC

NN (ours)	Exp.	SDF	0.9641	0.9986	0.9618	0.9988
NN (ours)	AF	SDF	0.9267	0.9958	0.9197	0.9968
NN (ours)	Exp.	CF	0.8164	0.9907	0.8067	0.9920
NN (ours)	AF	CF	0.7710	0.9891	0.7686	0.9904
AlphaFold2	-	-	0.8316	0.9561	0.8473	0.9643
DeepHelicon	-	-	0.5678	0.9336	0.5678	0.9366

**Table 4:** Individual sequences improved (in terms of Average Precision) - held out datasets.

Dataset	# Seqs (% of total)

TEST (49)	48 (98.0)
PREVIOUS (34)	33 (97.1)

**Table 5:** Feature mean and variance of Alphafold predicted and experimental structures.

		TRAIN		TEST		PREVIOUS	
Structure source	Features	Feature Mean	Feature Variance	Feature Mean	Feature Variance	Feature Mean	Feature Variance

Exp	SDF	−0.1616	0.3656	−0.2487	0.3363	−0.1634	0.3653
AF	SDF	−0.1744	0.3707	−0.2655	0.3338	−0.1979	0.3707
Exp	CF	−0.1716	0.3025	−0.2293	0.3466	0.0275	0.2969
AF	CF	0.0351	0.1604	0.1510	0.2799	0.0132	0.2474

Exp - Experimentally derived structures

AF - AlphaFold predicted structures

SDF - Structurally derived features

CF - Coordinates as features

## Footnotes

1
RCSB.org

3 Adapted from [60]
